# Necroptosis, tumor necrosis and tumorigenesis

**DOI:** 10.15698/cst2020.01.208

**Published:** 2019-12-19

**Authors:** Zheng-gang Liu, Delong Jiao

**Affiliations:** 1 Laboratory of Immune Cell Biology, Center for Cancer Research, National Cancer Institute; National Institutes of Health, 37 Convent Drive, Bethesda, MD 20892.

**Keywords:** necroptosis, tumor necroptosis, tumorigenesis, tumor metastasis, inflammation, immunosuppression

## Abstract

Necroptosis, known as programmed necrosis, is a form of caspase-independent, finely regulated cell death with necrotic morphology. Tumor necrosis, foci of necrotic cell death, occurs in advanced solid tumors and is often associated with poor prognosis of cancer patients. While it is well documented that apoptosis plays a key role in tumor regression and the inactivation of apoptosis is pivotal to tumor development, the role of necroptosis in tumorigenesis is still not fully understood as recent studies have reported both tumor-promoting and tumor-suppressing effects of necroptosis. In this short review, we will discuss some recent studies about the role of necroptosis in tumorigenesis and speculate the implications of these findings in future research and potential novel cancer therapy targeting necroptosis.

## INTRODUCTION

Cell death can happen through either an active, regulated process, known as programmed cell death, or a passive, uncontrolled course. Because of the dramatic morphological differences of these two types of cell death, they were originally coined as apoptosis and necrosis, respectively [1–4]. Apoptosis is defined as programmed cell death characterized by the activation of caspases, which are cysteine proteases that cleave cellular substrates, and the morphological features of cellular shrinkage, chromatin condensation, nuclear fragmentation, and membrane blebbing [1, 4]. At the end of the apoptotic process, dying cells are broken to membrane-bounded bodies containing the cellular structures and organelles, known as apoptotic bodies, which are taken up by surrounding cells or by phagocytic cells of the immune system without triggering inflammation [3, 4]. In contrast, necrosis is thought to be independent of the activity of caspases and is characterized by cellular swelling, organelle dysfunction, extensive mitochondrial damage, and plasma membrane rupture [2–4]. Because necrotic cells release their cell contents including proteins and nucleic acids, necrosis is much more inflammatory compared to apoptosis [2, 3].

In recent years, the concept of cell death has evolved dramatically because of the extensive studies of the role of cell death in normal tissue homeostasis and in the wide spectrum of diseases including autoimmune disease, neurodegenerative diseases and cancer [5, 6]. It is now accepted that there are other forms of programmed cell death such as pyroptosis and ferroptosis that are distinct from apoptosis [5, 6]. While engaging pyroptosis needs the activation of Caspase-1, pyroptotic cell death leads to the rupture of plasma membrane and the release of cell contents, which results in the subsequent inflammatory responses [5, 6]. Ferroptosis is a nonapoptotic, iron-dependent form of cell death [5, 6]. More importantly, it has been found that necrosis could also happen in a programmed, finely regulated fashion under certain conditions. For instance, when apoptosis is blocked, tumor necrosis factor (TNF) triggers certain types of cells to undergo a regulated necrotic cell death. This regulated necrosis is termed as necroptosis [5–9].

Apoptosis inhibits tumor progression and a hallmark of cancer is the ability of cancer cells to evade apoptosis [10]. Studies suggest that ferroptosis may play a similar role in tumorigenesis as apoptosis does [11]. While the important role of necroptosis in chemotherapy drug-induced cell death of cancer cells has been established, the role of necroptosis in tumorigenesis is still elusive. Here, we will go over some recent findings on the involvement of necroptosis in tumorigenesis and discuss the insights provided by these studies about the role(s) of necroptosis in tumor progression and the potential of novel cancer therapy targeting necroptosis.

## NECROPTOSIS: A TYPE OF PROGRAMMED NECROSIS

**Figure 1:**
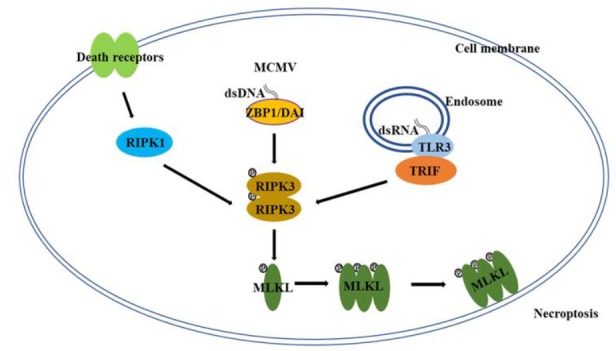
FIGURE 1: A simplified scheme of the molecular mechanism of necroptosis. In certain types of cells/tissues, necroptosis can be induced through different pathways under certain conditions including the engagement of death receptors and the infection of DNA or RNA viruses (MCMV: mouse cytomegalovirus). While RIPK3 and MLKL proteins are the core components of the necroptotic machinery, RIPK1, ZBP1/DAI, and TRIF are the key effectors that orchestrate the necroptotic process by recruiting RIPK3 in response to different stimuli. The aggregation of RIPK3 results in the autophosphorylation of the protein and the activated RIPK3 will phosphorylate MLKL and induce the oligomerization of MLKL, which then initiates the execution of necroptosis. DAI: DNA-dependent activator of interferon; MLKL: mixed lineage kinase domain-like; RIPK: receptor interacting protein kinase; TRIF: Toll-interleukin receptor-domain-containing adapter-inducing interferon-β; ZBP: Z-DNA-binding protein.

Necroptosis is a form of programmed, caspase-independent necrosis and has all of the morphological features of necrosis [5–9]. Necroptosis has originally been observed by studying death receptor-induced cell death [12], but it is clear now that necroptosis mostly happens under pathological conditions *in vivo*, such as viral infection [6–9]. Many aspects of the molecular mechanism of necroptosis were revealed through studying death receptor-induced necroptosis [6–9]. For death-receptor-induced necroptosis, the protein kinase receptor interacting protein kinase 1, 3 (RIPK1, RIPK3) and the mixed lineage kinase domain-like (MLKL) constitute the core of the necroptosis machinery **(Figure 1)** [6–9]. As a death domain containing kinase, RIPK1 plays a key role in multiple pathways of death receptor signaling, such as the activation of NF-κB and MAP (mitogen activated protein) kinases, and the induction of apoptosis and necroptosis [6–9, 13, 14]. Following the engagement of death receptors, RIPK1 is recruited to the death receptor signaling complexes through death domain interactions to mediate NF-κB and MAP kinase activation [13, 14]. Under certain conditions, these death receptor complexes could convert to the cytosolic, RIPK1-orchestered signaling complexes to mediate cell death. For instance, in the case of TNFR1 signaling, when the ubiquitination by cIAP (cellular inhibitor of apoptosis protein) proteins is inhibited, a RIPK1-centered cytosolic complex, known as complex IIa, is formed through recruiting FADD (Fas-associated death domain) and Caspase 8 (Casp-8) to trigger apoptosis [15–17]. When Casp-8 activity is blocked in certain types of cells, RIPK1 recruits RIPK3 to initiate the formation of complex IIb, also known as the necrosome, to initiate necroptosis [18–20]. It is important to point out that unlike its action in mediating the activation of NF-κB and MAP kinases, the pro-necroptotic role of RIPK1 requires its kinase activity and the death receptor-induced necroptosis could be blocked by inhibitors targeting RIPK1 kinase activity [21].

RIPK3 is another member of the RIP kinase family and lacks a death domain [22]. RIPK3 is recruited to the death receptor-induced necrosome by RIPK1 through their RIP Homotypic Interaction Motif (RHIM) interaction and is autophosphorylated in the necrotic complex [18–20]. Subsequently, the activated RIPK3 mediates the recruitment and the phosphorylation of MLKL protein [23, 24]. MLKL is the immediate downstream mediator of RIPK3 in necroptosis and is recruited to the necrosome by RIPK3 [23, 24]. As a pseudo kinase, MLKL contains N-terminal coiled-coil domains and a C-terminal kinase-like domain. After phosphorylation by RIPK3, MLKL oligomerizes through the N-terminal coiled-coil domains and translocates to the plasma membrane [25–28]. It has been suggested that MLKL mediates disruption of plasma membrane permeability by activating ion channels or forming pore structures directly in the plasma membrane [25–28]. Although the key events of necroptosis are believed to happen in the cytosol, interestingly, nuclear translocation of RIPK3 and MLKL has been observed and seems to play a role in accelerating the necroptotic process [29, 30].

Although RIPK1 plays an essential role of death receptor-mediated necroptosis, it is not required for viral infection- or Toll-like receptor (TLR)-triggered necroptosis [9]. Z-DNA-binding protein 1 (ZBP1), also known as DNA-dependent activator of interferon (IFN) regulatory factors (DAI), and Toll-interleukin receptor (TIR)-domain-containing adapter-inducing interferon-β (TRIF), two other RHIM domain-containing proteins, have been reported to function upstream of RIPK3-MLKL in viral infection- and TLR-induced necroptosis respectively **(Figure 1)** [31, 32]. Following viral infection or the ligation of pattern recognition receptors, ZBP1 and TRIF interact with RIPK3 through their RHIM domains to initiate the necroptotic process [31, 32]. While RIPK1, ZBP1 and TRIF are involved in different stimuli-induced necroptosis, apparently, there is some cross-talk among these proteins. For instance, it has been found that the necroptosis-inducing activity of ZBP1 is hindered by RIPK1 during normal embryonic development in a RHIM-dependent manner, because deletion of RIPK1 or the disruption of RIPK1 RHIM domain leads to ZBP1-dependent necroptosis and perinatal lethality [33, 34].

## NECROPTOSIS OF TUMOR CELLS AND TUMOR NECROSIS

Foci of cell death are commonly observed in core regions of solid tumors as a result of inadequate vascularization and subsequent metabolic stresses such as hypoxia and nutrient deprivation [35, 36]. Because the morphology of dead tumor cells appears to be necrotic, these foci of cell death are referred as tumor necrosis [37–39]. Tumor necrosis is often associated with aggressive tumor development and metastasis and is thought to be an indication of poor prognosis of patients with breast, lung and kidney cancer [38, 39]. However, the exact role of tumor necrosis in tumor development and metastasis remains elusive, because there is no available experimental system to manipulate necrotic cell death in tumors due to the lack of knowledge about the molecular mechanism of necrosis. Even so, some recent studies have started to shed light on the active role of tumor necrosis in tumorigenesis. Unlike apoptosis in which cells have intact membranes and are rapidly removed by host macrophages, it has been shown that tumor necrosis leads to the release of intracellular components to the tumor microenvironment [2, 3]. A recent study found that a high level of potassium was released from necrotic tumor cells [40]. Importantly, this study suggests that the extracellular potassium released from tumor necrosis inhibits both CD4 and CD8 T cell activities that are critical for anti-tumor immunity [40].

To explore the possible molecular mechanism of tumor necrosis, we recently tested if the necroptotic pathway is involved in tumor necrosis. As shown in our publication, we found that MLKL phosphorylation happens in dying cells in tumor necrotic areas and demonstrated that necroptosis is indeed engaged during tumor necrosis [41]. More importantly, tumor necrosis is largely suppressed upon necroptosis blockage by the deletion of MLKL gene and interestingly, the remaining tumor death in MLKL-null tumors is apoptotic [41]. These findings suggest that necroptosis of tumor cells is most likely the major cause of tumor necrosis and provides a feasible tool to assess the role of tumor necrosis in tumorigenesis [41].

Angiogenesis is critical for tumor growth. However, when solid tumors reach a certain size, scant vascularization happens in the core regions of advanced tumors and results in tumor necrosis [35, 36]. In these tumor areas with inadequate vascularization, tumor cells experience metabolic stresses such as hypoxia and nutrient deprivation [35, 36]. Previous studies have shown that metabolic stresses such as hypoxia, glutamine- or glucose-deprivation could trigger apoptotic, autophagy-dependent or necrotic cell death in cancer cells [42, 43]. However, whether these metabolic stress trigger necroptosis in cancer cells is still poorly studied. For example, a recent study suggested that GD (glucose deprivation?) triggers a RIPK1-dependent, non-necroptotic cell death [44]. Therefore, although these metabolic stresses are thought to be responsible for tumor necrosis, it is still not known whether these conditions indeed trigger necroptosis in solid tumors. This knowledge will help to fully understand the mechanism of tumor necrosis and its role in tumorigenesis.

## NECROPTOSIS IN TUMORIGENESIS

### A dual role in cancer development?

Evasion of programmed cell death is a hallmark of cancer and central to tumor development [10]. As a programmed necrosis, the role of necroptosis in tumor development has been investigated in the last few years. However, because both tumor suppressing and promoting effects have been reported, the role of necroptosis in tumor development is still not fully understood [45]. Several groups reported that RIPK3 expression is inhibited in many tumor cell lines and several types of cancer [46–48]. Restoring RIPK3 expression with the DNA methyl-transferase inhibitor (hypomethylation agent 5-aza-2'-deoxycytidine) potentiates the tumoricidal effects of the chemotherapy drug doxorubicin by inducing necroptosis [46]. More importantly, because necroptotic cell death triggers inflammatory responses, the immune-boosting effect of necroptosis will increase the anti-tumor immunity in the tumor microenvironment [49]. Particularly, the massive acute necroptosis by chemotherapy or irradiation has been shown to elevate anti-tumor immunity [49]. For instance, it has been shown that by releasing pro-inflammatory mediators such as high mobility group box 1 (HMGB1), radiation-induced tumor cell necroptosis may boost anti-tumor immunity and improve prognosis [50]. Interestingly, another report demonstrated that necroptotic cell death triggers robust cross-priming of cytotoxic CD8+ T cells in a RIPK1 and NF-κB-dependent manner, whereas the release of damage-associated molecular patterns (DAMPs) from dead cells alone is not sufficient [51]. This report reveals the critical role of the inflammatory pathway activated in dying cells in promoting anti-tumor immunity [51]. Therefore, these studies suggest that necroptosis may play a tumor suppressor role during tumor development.

Meanwhile, several other studies suggest that necroptosis promotes tumor growth [52, 53]. The study by Seifert *et al.* reported that necroptosis may have a promoting effect on tumor progression as the key players of necroptosis, RIPK1 and RIPK3, are critical for tumor development [52]. In pancreatic ductal adenocarcinoma (PDA), the necroptotic pathway was found to promote oncogenesis through releasing the chemokine (C-X-C motif) ligand 1 (CXCL1) and 130 kDa Sin3-associated polypeptide (SAP-130), which induce immune-suppression in the tumor microenvironment [52]. In this study, the authors found that CXCL1 is released from PDA cells in a RIPK3-dependent manner and CXCL1 blockage induces tumor regression by reducing the infiltration of immune suppressive MDSCs (myeloid-derived suppressor cells) and M2-like macrophages [52]. Meanwhile, the nucleus factor SAP-130 binds to Macrophage inducible Ca2+-dependent lectin receptor (Mincle), which is expressed on the cell surface of macrophages, and subsequently, those engaged macrophages with Mincle ligation suppress cytotoxic T cell infiltration and activation [52]. Although this study demonstrated the promoting effect of RIPK1 and RIPK3 in tumor development, necroptosis of tumor cells during tumor progression was not investigated. Most recently, we found that necroptosis happens in tumor necrotic areas and that blocking necroptosis switches tumor necrosis to tumor apoptosis [41]. Importantly, as necroptosis only happens in the late stage of tumor development in the breast cancer model used in our study, we found that inhibition of necroptosis reduces the late stage tumor growth and has no effect on tumor initiation and early growth [41]. Interestingly, while consistent with previous findings, we found that RIPK3 expression is decreased in the early stages of mouse MMVT-PyMT breast tumors, a significant increase of RIPK3 expression was detected in late stage tumors that bear tumor necrosis [41]. We also found that MLKL expression was significantly increased as well [41]. These findings suggest that the expression of these key necroptosis mediators was likely reprogramed to restore the necroptotic machinery in tumor cells when tumors experience metabolic stress. Taken together, the above discussed reports suggested the promoting role of necroptosis in tumor development. The finding that the elevated level of extracellular potassium from tumor necrosis suppresses the anti-tumor immunity supports this notion [40].

**Figure 2:**
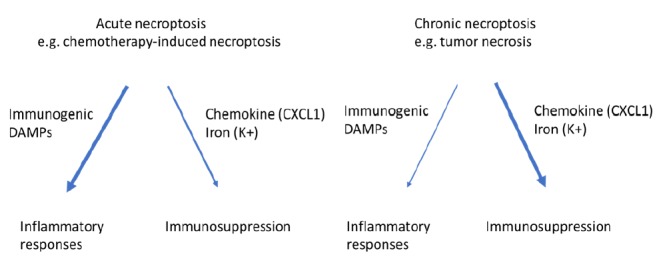
FIGURE 2: Necroptosis of tumor cells can have immunogenetic or immunosuppressive effect on tumor immunity. While acute necroptosis is more immunogenetic and favors anti-tumor immunoactivity, chronic necroptosis promotes pro-tumor immunity through releasing immunosuppressive factors such as CXCL1 and K^+^.

It is believed that the anti-tumor function of tumor immunity is mainly achieved by antigen specific, IFN-γ-expressing T cells, whereas the MDSCs, M2-like macrophages and regulatory T cells are the main components of the immune suppressive machinery [53, 54]. It is possible that when massive acute necroptosis happens, for example, following chemotherapy or irradiation treatment, boosting the anti-tumor immunity through activating IFN-γ-expressing T cells will be the dominant effect of necroptosis on tumor immunity **(Figure 2)**. In contrast, when mild chronic necroptosis happens, such as tumor necrosis, the dominant effect of necroptosis on tumor immunity will be immune suppression through consistently releasing immune suppressive molecules to modulate the tumor microenvironment including MDSC and M2-like macrophages **(Figure 2)**. These possibilities are consistent with the conclusion that necroptosis most likely plays a dual role in tumor development. However, further studies are necessary to fully understand the exact role of necroptosis of tumor cells in different stages of tumorigenesis.

### A critical facilitator in tumor metastasis?

Metastasis is common in patients with advanced cancer and is the main cause of cancer patient mortality [55, 56]. Although tremendous effort has been made to study metastasis [55], our knowledge about cancer metastasis is still quite limited for developing effective cancer therapies targeting metastasis [56]. Improving our understanding about the mechanism of tumor metastasis will help to identify novel therapeutic targets for containing metastasis. Because alternations of the tumor microenvironment play a critical role in metastasis [55, 56], and, as discussed above, chronic necroptosis has a profound effect on tumor microenvironments, we investigated the effect of blocking necroptosis on metastasis in our recent study [41]. In the orthotopic MVT-1 breast cancer model, we found that blocking necroptosis of tumor cells by MLKL deletion significantly inhibited lung metastasis [41]. We confirmed that necroptosis inhibits the anti-tumor activity of T cells as reported by earlier studies [40, 52] (unpublished data). Interestingly, the depletion of CD8+ T cells only partially reduced the difference of tumor metastasis between wild tpe and MLKL knock out tumor in the MVT-1 model (unpublished data), indicating that, in addition to inhibiting the anti-tumor activity of T cells, necroptosis likely modulates other aspects of the tumor microenvironment to promote metastasis.

Consistent with this possibility, our study on the regulation of necroptosis showed that a known metastasis promoting factor, soluble E-cadherin (sE-Cadherin), is released from the necroptotic cell surface [57]. We found that the cell-surface proteases, A Disintegrin And Metalloproteases (ADAMs), are activated at the very early stage of necroptosis and that all cell-surface proteins including E-cadherin are cleaved by ADAMs [57]. It is known that sE-Cadherin promotes tumor cell invasion and metastasis by interfering with intercellular adhesion junction, increasing matrix metalloproteinases activity, and enhancing angiogenesis [58]. In addition, chemokine CXCL-1 is another necroptosis-elevated molecule that is known to promote tumor metastasis [52, 59]. Currently, we are investigating whether those proteins released/increased by necroptosis play a key role in tumor metastasis in spontaneous cancer models in which necroptosis is specifically abolished in tumor cells. Based on clinical observations, tumor necrosis of solid tumors has long been considered as an indication of metastatic tumors [37–39]. Linking necroptosis of tumor cells with tumor necrosis provides a tool to demonstrate the important role of tumor necrosis in metastasis experimentally and provides a potential novel target for intervening this deadly event of tumorigenesis.

Interestingly, not only necroptosis of tumor cells has the promoting effect on tumor metastasis, necroptosis in other types of cells also plays a key role in advancing metastasis. It is reported that circulating tumor cells induce necroptosis of endothelial cells through engaging death receptor 6 to promote tumor cell extravasation and metastasis [60]. Importantly, the specific inhibitor of RIPK1, necrostatin-1, could efficiently reduce tumor cell extravasation and metastasis [60]. Therefore, targeting necroptosis in general may be an effective novel therapy for restraining metastasis.

## NECROPTOSIS AND TUMORIGENESIS IN PERSPECTIVE

While recent literature clearly demonstrates the involvement of necroptosis in numerous aspects of tumorigenesis, many fundamental questions regarding the regulation and the role of necroptosis in tumorigenesis remain elusive. Here are a few of examples:

1) what signal(s) triggers necroptosis in tumor cells during tumor development? Do death factors TNF, FasL (Fas ligand) and TRAIL (TNF related apoptosis inducing ligand) play a role in the induction of tumor necroptosis? Although it has been suggested that death receptors-induced necroptosis may play a role in inflammation-driven tumor initiation and development, it has not been demonstrated experimentally that these death factors indeed trigger necroptosis of tumor cells during tumorigenesis.

2) Does the tissue specificity of different types of tumors affect the role of tumor necroptosis in tumorigenesis? So far, the role of necroptosis in tumorigenesis was only tested in limited types of cancers such as PDA and breast cancer [41, 52]. The role of necroptosis in tumorigenesis needs to be examined in other types of solid tumors as well as “liquid tumors” (blood cancers). Interestingly, a recent study suggests that necroptosis plays a key role in liver cancer lineage commitment [61].

3) Is the evasion of necroptosis a critical event for tumor initiation or early development? The findings that RIPK3 expression is silenced in many types of tumor cell lines and cancers and that necroptosis augments the anti-tumor activity of T cells leads to the speculation that necroptosis inhibition may be a key event for tumor development in some types of cancers [46–48, 51]. However, this notion still needs to be evaluated experimentally in cancer models.

4) What are the underlying mechanisms for the promoting effect of tumor necroptosis on tumor development and metastasis in addition to modulation of tumor immunity? Considering the profound effect of tumor necrosis on tumor microenvironments, it is important to investigate what other component(s) of tumor microenvironments in addition to tumor immunity are altered to promote tumor growth and metastasis.

Finally, 5) what is the long-term effect of chemo-/radiation-induced necroptosis on tumor growth and metastasis? The immunogenic effect of acute massive necroptosis is clearly beneficial for chemo- or radiation-induced initial tumor regression. However, since chronic necroptosis could promote tumor growth and metastasis, will necroptosis by repeated chemo- or radiation treatments be more anti- or pro-tumorigenesis in the long haul?

Addressing these questions will greatly improve our understanding about the role of necroptosis in tumorigenesis and provide new insights about the possibility and effectiveness of targeting necroptosis as a cancer therapy.

## Abbreviations:

ADAM: – A Disintegrin And Metalloproteases,
Casp-8: – caspase 8,
CXCL: – (C-X-C motif) ligand,
IFN: – interferon,
MAP: – mitogen activated protein,
MDSC: – myeloid-derived suppressor cell,
Mincle: – macrophage inducible Ca2+-dependent lectin receptor,
MLKL: – mixed lineage kinase domain-like,
PDA: – pancreatic ductal adenocarcinoma,
RHIM: – RIP Homotypic Interaction Motif,
RIPK: – receptor interacting protein kinase,
SAP-130: – 130 kDa Sin3-associated polypeptide,
sE-Cadherin: – soluble E-Cadherin,
TLR: – toll-like receptor,
TNF: – tumor necrosis factor,
ZBP: – Z-DN- binding protein.
